# Luminescence intensity of vortex cavitation in a Venturi tube changing with cavitation number

**DOI:** 10.1016/j.ultsonch.2020.105389

**Published:** 2020-11-13

**Authors:** Hitoshi Soyama

**Affiliations:** Department of Finemechanics, Tohoku University, 6-6-01 Aramaki, Aoba-ku, Sendai 980-8579, Japan

**Keywords:** Luminescence, Hydrodynamic cavitation, Vortex, Sound velocity, Pressure

## Abstract

Hydrodynamic cavitation in a Venturi tube produces luminescence, and the luminescence intensity reaches a maximum at a certain cavitation number, which is defined by upstream pressure, downstream pressure, and vapor pressure. The luminescence intensity of hydrodynamic cavitation can be enhanced by optimizing the downstream pressure at a constant upstream pressure condition. However, the reason why the luminescence intensity increases and then decreases with an increase in the downstream pressure remains unclear. In the present study, to clarify the mechanism of the change in the luminescence intensity with cavitation number, the luminescence produced by the hydrodynamic cavitation in a Venturi tube was measured, and the hydrodynamic cavitation was precisely observed using high-speed photography. The sound velocity in the cavitating flow field, which affects the aggressive intensity of the cavitation, was evaluated. The collapse of vortex cavitation was found to be closely related to the luminescence intensity of the hydrodynamic cavitation. A method to estimate the luminescence intensity of the hydrodynamic cavitation considering the sound velocity was developed, and it was demonstrated that the estimated luminescence intensity agrees well with the measured luminescence intensity.

## Highlights

• Vortex cavitation is important phenomenon in luminescence of hydrodynamic cavitation.
• Pressure wave induced by collapse of vortex cavitation is visualized.
• Sound velocity in cavitating flow field is evaluated by observing the pressure wave.
• The sound velocity is key factor of luminescence intensity.
• Luminescence intensity is enhanced by optimizing pressure where bubble collapse.

## Graphical abstract

**Figure:**
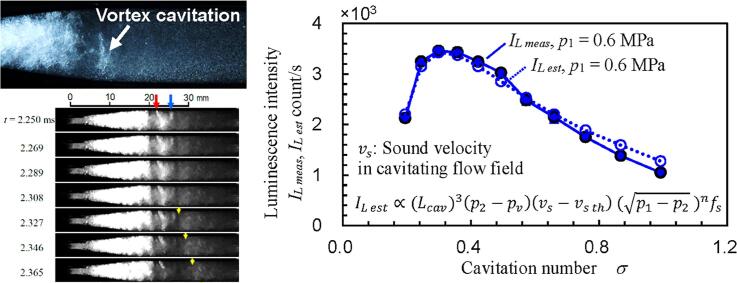

## 1 Introduction

**Fig. 1:**
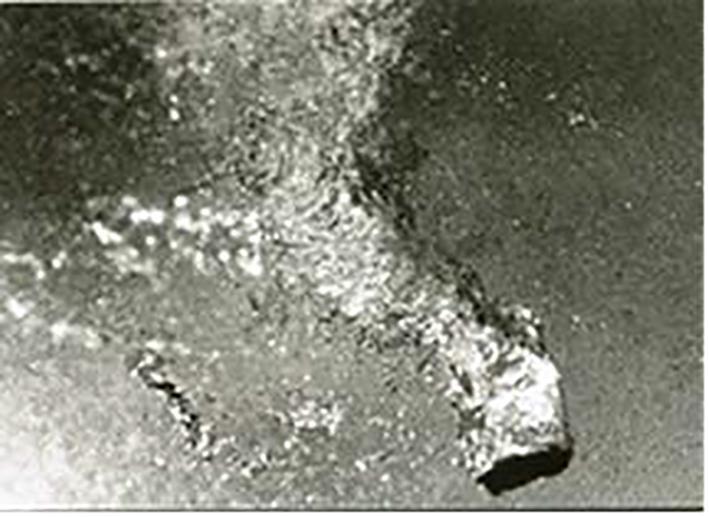
Typical vortex cavitation [3]

Cavitation in fluid machineries, i.e., hydrodynamic cavitation, has a severe impact on bubble collapse, and cavitation causes severe erosion in hydraulic components, such as pumps [1], [2] and valves [3]. However, the impact can be utilized for mechanical surface enhancement to enhance material properties, such as fatigue strength, in the same way as shot peening [4], [5], and this is called “cavitation peening” [6]. Through research on cavitation damage and cavitation peening, Soyama et al. found that vortex cavitation, as shown in Fig. 1, produced a severe impact [3], and vortex cavitation occurring in the Venturi tube produced luminescence [7]. Vortex cavitation is initiated from the cavitation nuclei, which become longitudinal bubbles because of low pressure at the vortex core. The vortex, which produces vortex cavitation, is a type of turbulent eddy. It was reported that the aggressive intensity of hydrodynamic cavitation in a Venturi tube was enhanced by approximately 100 times when the pressure was increased in the region where the bubble collapses without additional power. Thus, it is worthwhile to clarify the enhancement mechanism of the aggressive intensity of vortex cavitation in a Venturi tube.

Since the discovery of abrupt cavity separations on a cavitating hydrofoil [8], many researchers have focused on cloud cavitation [9], [10], [11], [12], [13], [14]. As Arndt reviewed [12], cloud cavitation is a form of cavitation in vortical flows. Whereas a typical vortex cavitation is “tip vortex cavitation,” which occurs in the tip vortex of a screw propeller [15], in the present work, “vortex cavitation” is used to describe the cavitation, as shown in Fig. 1. The vortex cavitation as shown in Fig. 1 was observed in the shear flow region downstream of the butterfly valve. In Fig. 1, the main flow direction was from the right-hand side to the left-hand side. The vortex cavitation was developed at the boundary between the main flow region, i.e., the orifice jet, and the recirculating region. More details are given in reference [16]. A video regarding vortex cavitation is available in the electronic version as supplementary data.

In the case of hydrodynamic cavitation, a practical application for wastewater treatment has been proposed using multiple orifices [17], [18], [19], [20], [21], [22], [23], [24]. Disruption of *Escherichia coli* [25], degradation of dichlorvos [26], removal of pharmaceuticals from wastewater [27], waste-activated sludge pretreatment [28] and effluent treatment [29] have also been proposed. Regarding the treatment of biomass using hydrodynamic cavitation, treatment of cellulose [30], [31], [32], delignification of wheat straw [33], pretreatment of lignocellulosic biomass [34], pretreatment of rice bran for microbial fuel cells for electricity generation [35], pretreatment of sugarcane bagasse [36], biomass extraction [37] and energy harvesting with microscale hydrodynamic cavitation thermoelectric generation coupling [38] have been investigated. Pretreatments of biomass using hydrodynamic cavitation and ultrasonic cavitation were compared [34]. In the experiment, ultrasonic cavitation was produced by a probe-type ultrasonic generator (Sonifier 250, Branson Ultrasonics) with a vibration tip (12.7 mm extension) fixed in a glass beaker with an inner diameter of 4.7 cm and height of 9.0 cm, in which the biomass powder (2.0) g was suspended in 50 mL of solution. The frequency was 20 kHz, ultrasonic power output was 50 W, and treatment time was 1 h. The estimated obtained glucose was 0.196 g. By contrast, hydrodynamic cavitation was produced using a diaphragm pump (Duplex II D3635E7011A, FLOJET), that was driven by a 30 W motor. The biomass powder (16 g) was suspended in 400 mL of solution and treated for 1 h. The obtained glucose estimated through hydrodynamic cavitation was 2.42 g. Thus, the pretreatment efficiencies of ultrasonic cavitation and hydrodynamic cavitation were 0.109 × 10^-5^ g glucose/J and 2.24 × 10^-5^ g glucose/J, respectively. Hence, it was concluded that the efficiency of pretreatment using hydrodynamic cavitation was 20 times better than that of ultrasonic cavitation [34]. Other applications using hydrodynamic cavitation, such as nanosuspensions [39], biodiesel production [40], generation of pharmaceutical nanoemulsions [41], degradation of carbamazepine [42], increase in biochar surface area and porosity [43], ammonia stripping [44], liposome destruction [45] and deactivation of *Salmonella typhimurium* [46], have been proposed.

Because it was reported that the aggressive intensity of hydrodynamic cavitation was enhanced by an increase in the pressure in the bubble collapse region [7], the issue of cavitation number value in studies of water treatment by hydrodynamic cavitation was raised [47]. From the viewpoint of cavitation erosion caused by hydrodynamic cavitation, the erosion rate was maximum at a certain cavitation number [48], [49]; however, the reason for this is not clear. Although it is known that there is a correlation between the acoustic impedance and cavitation erosion rate [50], [51], sound velocity in the cavitating flow field has hardly been investigated. The propagation of sound through a liquid containing bubbles [52] and its sound velocity [53] were investigated, and they were changed by the diameter of the bubbles [54]. It was also reported that the sound velocity depended on the void fraction, and it is lower than the sound velocity in air or liquid. [55]. Thus, in the present study, the sound velocity in the cavitation flow field, which affects the aggressive intensity of the cavitation, was evaluated.

Cavitation has been studied for a long time, spherical bubbles have been investigated mainly by simulations [56] and experimental studies [57], [58], [59], and the effects of bubble shape and bubble interactions have also been studied [60]. Furthermore, the collapse of spherical cloud cavitation consisting of fine bubbles was analyzed numerically [61]. Hydrodynamic cavitation was also investigated theoretically [62] for cloud cavitation; however, vortex cavitation was not investigated. Although modeling of hydrodynamic cavitation with an orifice or in a Venturi tube was proposed [63], [64], only shear flow was considered. Such vortices as Rankine's vortex cavitation were not considered. The behavior of the vortex cavitation shown in Fig. 1 can be explained by considering Rankine's vortex [65]. A vortex-based cavitation device was proposed and simulated numerically [66]; however, the vortex used was similar to a suction vortex, which is observed in hydro turbines or pumps. It is different from the target vortex, which is closely related to a turbulent eddy. Although the vortex structure of cavitating flow is important in cavitation peening [4], [5], [6], [67], there is significant misunderstanding of the vortex flow around the submerged water jet [68], [69], [70], and a spherical bubble in shear flow was assumed in the simulation [71], [72].

From the viewpoint of luminescence, as Suslick and Flannigan reviewed [13], much research on the luminescence caused by a collapse of spherical bubbles has been reported [73], [74], and photons have been reported to be proportional to the maximum diameter of the bubble [75]. In the cases of ultrasonic cavitation and hydrodynamic cavitation, it was believed that the chemical effect of cavitation was caused by a collapse of the spherical bubble [76]. However, in the case of hydrodynamic cavitation, experimental results on the oxidation of aqueous KI solution with a hydrodynamic cavitation setup [77], hydrogen produced by a cavitating jet [78], luminescence on a hydrofoil [79], [80], luminescence caused by a cavitating jet [81], luminescence arising in a Venturi tube [7] and luminescence in the microchannel [82], [83] were obtained. Although a cluster approach was proposed [84], [85], the shape was spherical. It was reported that there is an effect of the shape, such as rectangular and circular, when comparing the shapes of Venturi tubes [86], but flow was not observed. Although a method of hydrodynamic cavitation using multiple orifices was proposed [87], it may lead to a reduction in processing efficiency, because the vortex structure is miniaturized. From the viewpoint of luminescence [7], [60], [88], [89] and erosion [90], the effect of the sound velocity of dissolved gas has been considered. There has been no report on evaluating the sound velocity of the cavitating flow field itself. The issue of studying cloud cavitation was proposed [91], but no systematic studies have been conducted on the luminescence of vortex cavitation and the evaluation of the sound velocity of the cavitating flow field.

The Weissler reaction has been used to measure the aggressive intensity of ultrasonic and hydrodynamic cavitation, however, it had been reported that the Weissler reaction is not a good model reaction for assessing the effectiveness of hydrodynamic cavitation [92]. Moreover, it has been demonstrated that the luminescence intensity of hydrodynamic cavitation is related to the aggressive intensity [7], [93]. In addition, considering the results of the luminescence spectra and the electron spin resonance ESR spectra of hydrodynamic cavitation [7], [93], the luminesces of hydrodynamic cavitation in the Venturi tube is mainly caused by hydroxyl radicals. As is well known, the radicals would assist the chemical reactions.

In the present study, vortex cavitation in a Venturi tube, which produced luminescence, was precisely investigated through high-speed photography. In addition, the sound velocity in a cavitating flow field was evaluated because sound velocity is one of the most important factors affecting the aggressive intensity of hydrodynamic cavitation. An estimation method for the luminescence intensity produced by hydrodynamic cavitation was developed considering the sound velocity. It was demonstrated that the estimated luminescence intensity had a good correlation with the measured luminescence intensity.

## 2 Experimental apparatus and procedures

**Fig. 2:**
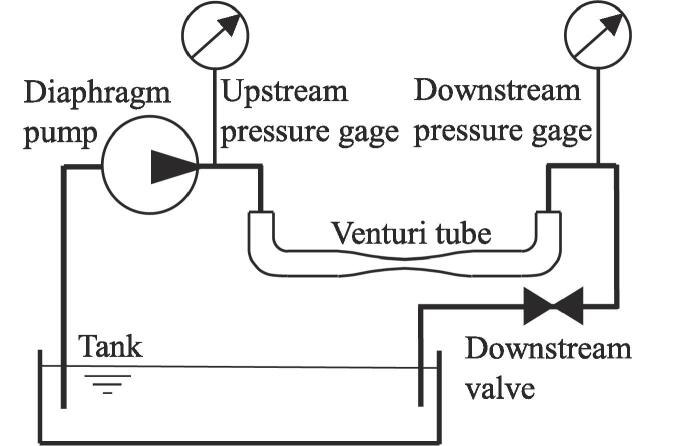
Schematic diagram of experimental apparatus.

**Fig. 3:**
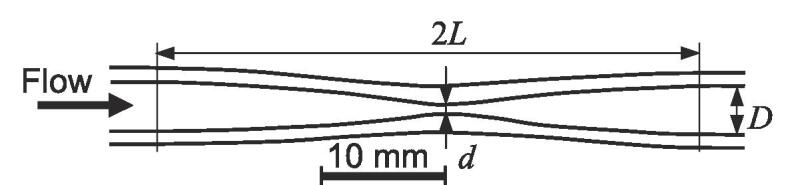
Schematic diagram of test section (*d* = 1.2 mm).

Fig. 2 is a schematic diagram of a hydrodynamic cavitation system. The ion-exchanged water was poured into a tank, pressurized by a diaphragm pump, and then injected into a Venturi tube made of quartz glass. The temperature of the tested water was 293 ± 2 K. The downstream pressure of the Venturi tube was controlled by a downstream valve. The upstream pressure *p*_1_ and the downstream pressure *p*_2_ of the throat of the Venturi tube were measured using upstream and downstream pressure gauges. In the present study, the absolute pressures were used for *p*_1_ and *p*_2_, because the target phenomenon was cavitation. This means that, when the downstream pressure is atmospheric pressure, *p*_2_ is 0.1 MPa. The geometry of the Venturi tube used is shown in Fig. 3. The inner diameter *D* of the tube was 3.6 mm and throat diameter *d* of the Venturi tube was 1.2 mm, as previously reported [7]. The length of the curved area, 2*L*, was 40 mm.

The aspect of hydrodynamic cavitation in the Venturi tube was observed by a high-speed video camera, whose maximum recording speed was 4000 frames per second (fps) in the full frame and 109,999 fps in the partial frame. The aspect was also taken by instantaneous photography using a digital camera with a flush lamp whose exposure time was 1.1 μs. The high-speed video camera and the digital camera were placed perpendicular to the Venturi tube. Because the shedding of vortex cavitation is a periodic phenomenon, the shedding frequency was evaluated by image analysis [94] as follows. The brightness change with time at a certain point was obtained from the images captured by the high-speed video camera, and the data were analyzed through fast Fourier transform. The average value and standard deviation of the shedding frequency were calculated using three frequencies from the maximum power spectral density (PSD) to the third PSD. The image acquisition and analysis software used were Motion Studio ver. 2.15, IDT Inc. and Motion analyzer ver. 1.5, Keyence Corp., respectively. The recording speed and the number of analyzed frames of the high-speed video camera that was used to observe the unsteady behavior of vortex cavitation at the vortex shedding were 51,999 fps and 4,096 frames, respectively. The sampling rate was 1.92 × 10^-5^ s (=1/51,999) and the sampling length was 7.88 × 10^-2^ s (=1.92 × 10^-5^ × 4,096). Considering sampling theorem, the highest and the lowest frequencies associated with image acquisition and analysis of the unsteady behavior of vortex cavitation were 26.0 kHz (=51,999/2) and 12.7 Hz (=1/(7.88 × 10^-2^)), respectively.

The luminescence caused by hydrodynamic cavitation was evaluated using a luminescence analyzer. The photomultiplier tube in the analyzer can detect 50–10^8^ photons/cm^2^/s. One count of the analyzer is equivalent to 50 photons. During the evaluation of the luminescence, the Venturi tube was set in the test chamber in the analyzer. It was reported that the luminescence spot was observed at the collapsing region of hydrodynamic cavitation [93]. In the horizontal direction, the throat of the Venturi tube was set 30 mm downstream from the center of the window for the photomultiplier. The vertical distance between the center of the Venturi tube and the window for the photomultiplier was 40 mm. When the cavitating region was decreased, the luminescence spots became further from the photomultiplier to verify that the shorter the cavitating length, the higher the luminescence intensity. In the present experiment, the measuring time of the luminescence was 10 s, and it was repeated six times under each condition. Then, the averaged value and standard deviation were calculated from the six data.

The cavitation number, *σ* which is a key parameter of the cavitating flow, is defined as follows:

(1) $$\sigma =\frac{p_{2}-p_{v}}{\frac{1}{2}\rho U^{2}}=\frac{p_{2}-p_{v}}{p_{1}-p_{2}}$$

Here, *p_v_* and *ρ* are the vapor pressure and density of the test water, respectively, and *U* is the velocity at the throat.

## 3 Results

### 3.1 Vortex cavitation in the Venturi tube

**Fig. 4:**
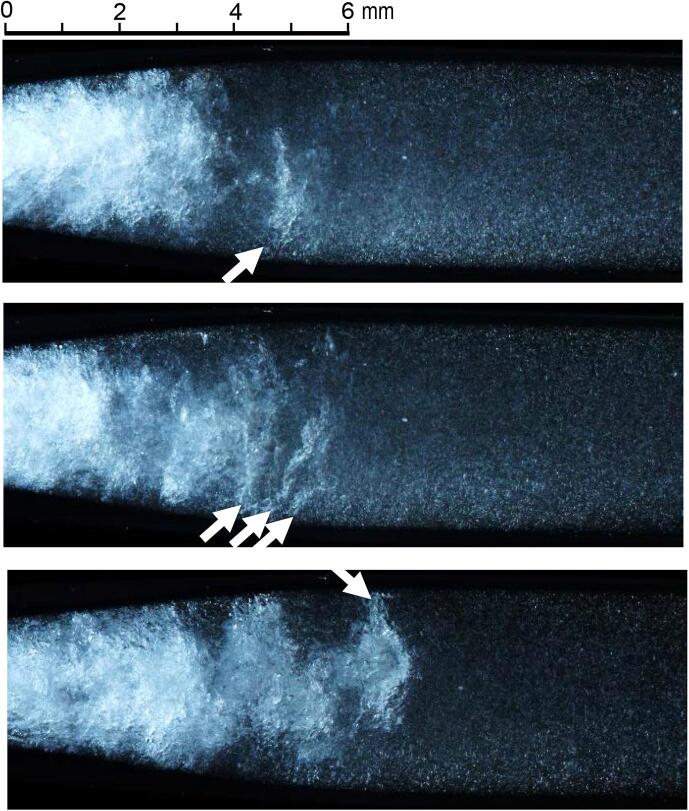
Typical aspects of vortex cavitation in Venturi tube (*p*_1_ = 0.6 MPa, *p*_2_ = 0.1 MPa).

Fig. 4 shows typical aspects of vortex cavitation in the Venturi tube. In Fig. 4, the flow direction is from the left-hand side to the right-hand side, and the dense white area is the cavitating region. Typical vortex cavitation is indicated by a white arrow in Fig. 4. Several vortex cavitations, whose vortex cores are visualized by longitudinal bubbles, as shown in Fig. 1, are observed at the downstream edge of the cavitating region. The length and the diameter of a typical vortex cavitation are several millimeters and submillimeter order, respectively. The dimmed extremely small bubbles downstream of the vortex cavitation are small air bubbles, and they are called “residual bubbles” [95] because they exist after cavitation bubble collapse.

**Fig. 5:**
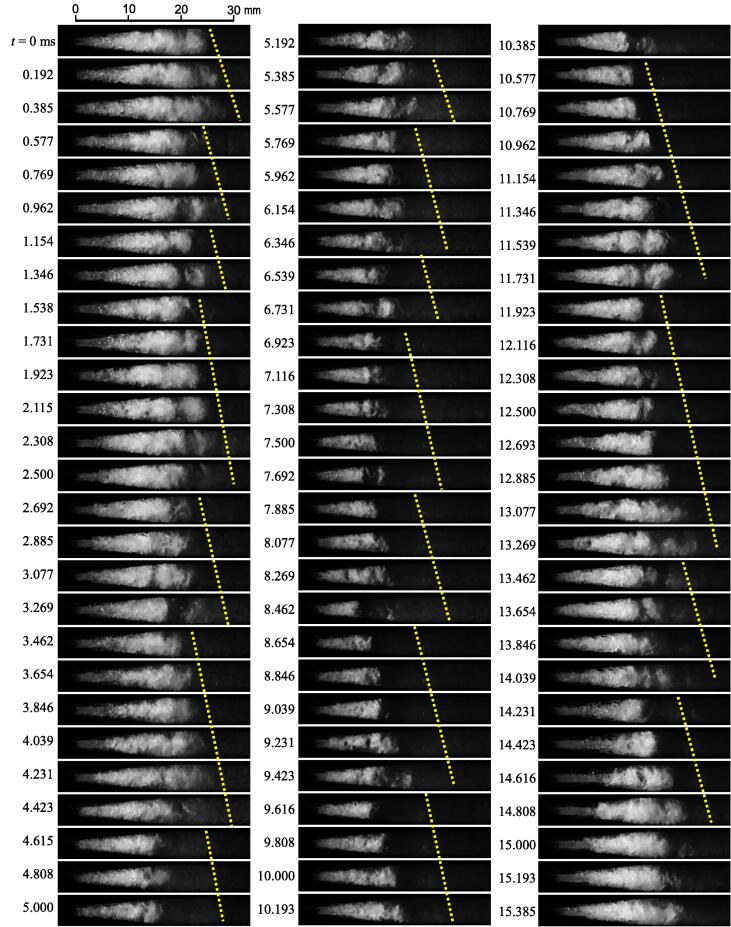
Unsteady behavior of vortex cavitation (*p*_1_ = 0.6 MPa, *p*_2_ = 0.1 MPa, *t* = 0 – 15.385 ms).

**Fig. 6:**
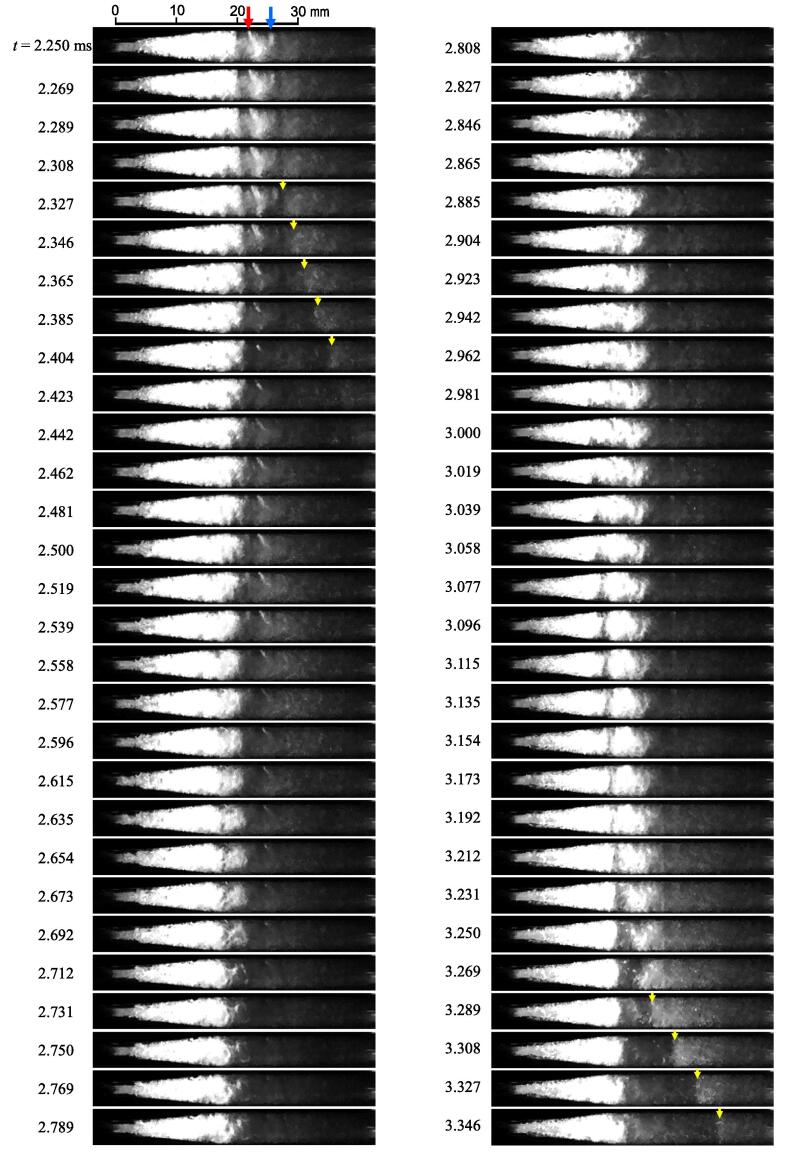
Unsteady behavior of vortex cavitation (*p*_1_ = 0.6 MPa, *p*_2_ = 0.1 MPa, *t* = 2.250 – 3.346 ms).

To demonstrate the unsteady behavior of the vortex cavitation, Fig. 5, Fig. 6 reveal the aspect of hydrodynamic cavitation at *p*_1_ = 0.6 MPa and *p*_2_ = 0.1 MPa in the Venturi tube as observed by the high-speed video camera. In Fig. 5, Fig. 6, the recording speed of the camera was 51,999 fps. In Fig. 5, Fig. 6, the flow direction is from the left-hand side to the right-hand side. The picture in Fig. 5 shows every 10 images of *t* = 0 –15.385 ms. As the cavitating length changes periodically [7], the yellow dotted line is placed at the downstream of the trailing edge of the cavitating region to investigate periodical frequency. The yellow dotted line was shifted slightly downstream to avoid disturbance of the cavitation aspect. As shown in Fig. 5, the trailing edge of the cavitating region was broken up, and then the vortex or cloud cavitation was shedding periodically downstream. For example, when the dense white region, i.e., the cavitating region, at *t* = 2.692 ms was observed, the cavitating length was about 22 mm and it increased with the time, and then it was broken up at 15 mm at *t* = 3.077 ms. The cloud cavitation at the trailing edge of the cavitating region was shedding downstream; it disappeared at *t* = 3.462 ms. When the images of the periodic phenomenon were counted, the shedding frequency of the vortex cavitation was approximately 0.65 – 2.6 kHz. For example, the cavitating region was increased with time in four pictures from *t* = 2.692, 5.769, 7.885, 13.462, and 14.231 ms. The four pictures in Fig. 5 mean 40 frames, because the picture in Fig. 5 shows every 10 images. The duration of 40 frames was 0.769 ms, and it was 1.3 kHz. Five patterns, i.e., 0.769 ms × 5 = 3.845 ms, were observed in 15.385 ms. Thus, the possibility of occurrence per unit time was 25%. The development of the cavitating region in five pictures and six pictures correspond to 1.04 and 0.87 kHz. The number of observations was three patterns for five pictures and two patterns for six pictures; thus, the possibility was 18.7% for five pictures and 15% for six pictures. Because the possibility of patterns of four to six pictures covered about 60%, the shedding frequency of vortex cavitation at *p*_1_ = 0.6 MPa and *p*_2_ = 0.1 MPa was 0.87 – 1.3 kHz.

As shown in Fig. 5, the cavitating length decreased with the periodic shedding from *t* = 0 ms to *t* ≈ 8 ms, and the cavitating length increased from *t* ≈ 8 ms to *t* ≈ 15 ms. For example, the cavitating length at *t* = 0.192 ms was approximately 25 mm, decreasing to approximately 10 mm at *t* = 7.5 ms and then increasing to 25 mm at *t* = 15.385 ms. In other words, the cavitating length changes at a frequency of approximately 60 Hz. At *p*_1_ = 0.6 MPa, as the diaphragm pump produced pressure fluctuations with an amplitude of approximately ± 10% and a frequency of 57.0 ± 2.5 Hz, the changes in the cavitating region were caused by the pressure fluctuation of the pump. The frequency of the vortex shedding was 10 or 20 times higher than that of the pump pressure fluctuation.

The behavior of vortex cavitation can be observed precisely using the images in Fig. 6. The pictures in Fig. 6 show each image at *t* = 2.250 – 3.346 ms in Fig. 5. To clarify the rebound of the vortex, in Fig. 6, the brightness is adjusted to be brighter than that in Fig. 5. The typical vortex cavitation at *t* = 2.250 ms is indicated by blue and red arrows. The vortex cavitation with the blue and the red arrow shrank at *t* = 2.289 and 2.404 ms and then rebounded. At both collapses, the velocity of the axial collapse was approximately 46 m/s because the axial length of the vortex was half the tube diameter, i.e., 1.8 mm, and they collapsed within two frames, i.e., 0.039 ms. As is clearly shown at *t* = 2.5 ms, the rebounded shape of the vortex cavitation was also a longitudinal bubble. The collapse and rebound of the real vortex cavitation were similar to the behavior of the modeled cavitating vortex generated by a rotating device [96].

The other interesting phenomenon in Fig. 6 is shown by the yellow arrows. After the shrinkage of the vortex cavitation indicated by the blue arrow, the boundary between the black and white regions shifted downstream at *t* = 2.346 – 2.442 ms. A similar phenomenon was observed at *t* = 3.289 – 3.346 ms. When the moving velocity of the boundary was calculated, it was 104 m/s at *t* = 2.346 – 2.442 ms and 187 m/s at *t* = 3.289 – 3.346 ms. Because the flow condition of Fig. 6 was *p*_1_ = 0.6 MPa and *p*_2_ = 0.1 MPa, the maximum flow velocity was 31.6 m/s. In other words, the moving velocity of the boundary was much larger than the maximum flow velocity because of the pressure difference. The details of the moving velocity of the boundary are discussed in Section 3.2.

**Fig. 7:**
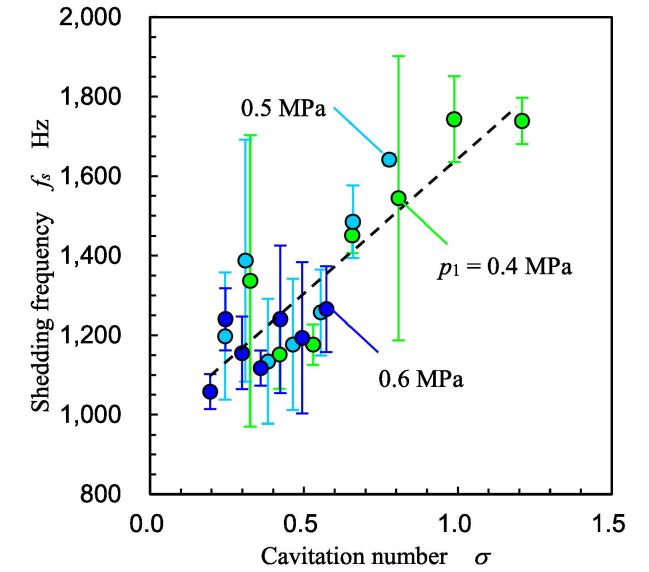
Shedding frequency of vortex cavitation.

In Fig. 6, a part of the periodic shedding is also observed. The large vortex cavitation collapsed at *t* ≈ 2.4 and 3.3 ms. The estimated cycle was 1.1 kHz, and it was between 0.87 and 1.3 kHz, as mentioned above. Fig. 7 illustrates the shedding frequency *f_s_* at various *p*_1_ and *p*_2_ values obtained by image analysis [94]. The *f_s_* obtained by the image analysis at *p*_1_ = 0.6 MPa and *p*_2_ = 0.1 MPa was 1.06 ± 0.04 kHz, and it was between 0.87 and 1.3 kHz. Thus, the *f_s_* obtained by the image analysis is reasonable. In Fig. 7, *f_s_* is shown as a function of cavitation number *σ* as the shedding frequency of hydrodynamic cavitation changes with *σ* [12], [97]. As the superharmonics and subharmonics of the shedding frequency were detected, the data points were scattered, and the standard deviation was large. For example, the shedding frequencies of the five and six pictures patterns in Fig. 5 were 1.04 kHz and 0.87 kHz, respectively. Furthermore, the frequencies of the two and three pictures patterns in Fig. 5 were 2.6 kHz and 1.7 kHz, respectively. Specifically, two and three pictures patterns are a type of superharmics of the five and six pictures patterns. By contrast, when the vortex cavitation did not reach the image analysis area, the subharmonics frequency was obtained from the FFT analysis. In any case, *f_s_* was roughly proportional to *σ*, and this tendency corresponds to *f_s_* ∝ *σ* ^0.83±0.10^, which was obtained from the observation of the cavitating jet [97]. As the vortex cavitations continue shedding downstream with each other as in the Karman vortex shedding, they become larger and larger. Thus, the shedding frequency decreases with an increase in the cavitating length, i.e., a decrease in the cavitation number. In other words, the shedding frequency increases with an increase in the cavitation number. This tendency is similar to the cloud shedding of the cavitating jet [97], [98]. Then, the experimental formula to estimate *f_s_* [Hz] is described by Eq. (2). Here, *c*_0_ is constant and equal to 1 Hz.

(2) $$f_{s}=c_{0}\left(688\sigma +961\right)$$

### 3.2 Sound velocity in the cavitating flow field

**Fig. 8:**
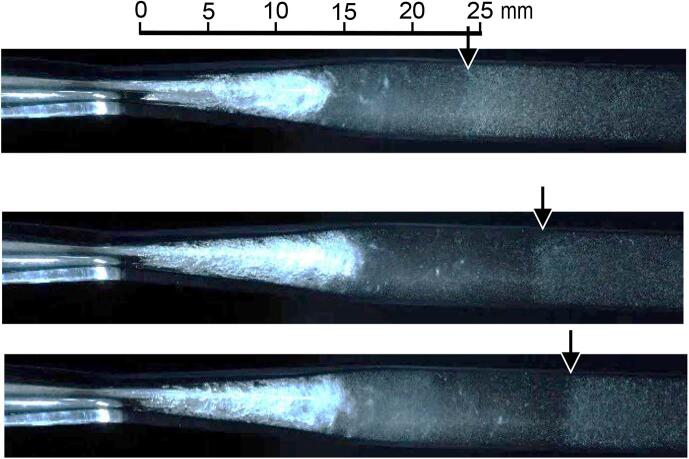
Typical aspects of visualized pressure wave by shrinkage of residual bubbles (*p*_1_ = 0.6 MPa, *p*_2_ = 0.1 MPa).

To investigate the phenomenon of the boundary between the black and white regions in Fig. 6, Fig. 8 shows the cavitating aspects taken by the instantaneous photography. The boundary indicated by the arrow was clearly observed. Many tiny residual bubbles were observed in the white region, and the residual bubbles were scarcely observed in the black region. As mentioned above, the moving velocity was much higher than the flow velocity, and the collapse of the vortex cavitation was observed at the starting time of the boundary movement in Fig. 6. Thus, this phenomenon suggests that the pressure produced by the vortex cavitation collapse propagated with the collapse of residual bubbles. After the pressure wave had passed, the residual bubbles collapsed, and residual bubbles were hardly observed. This would be the mechanism of the movement of the boundary between the black and white regions. Then, the movement velocity suggested the propagating velocity of the pressure, i.e., sound velocity.

**Fig. 9:**
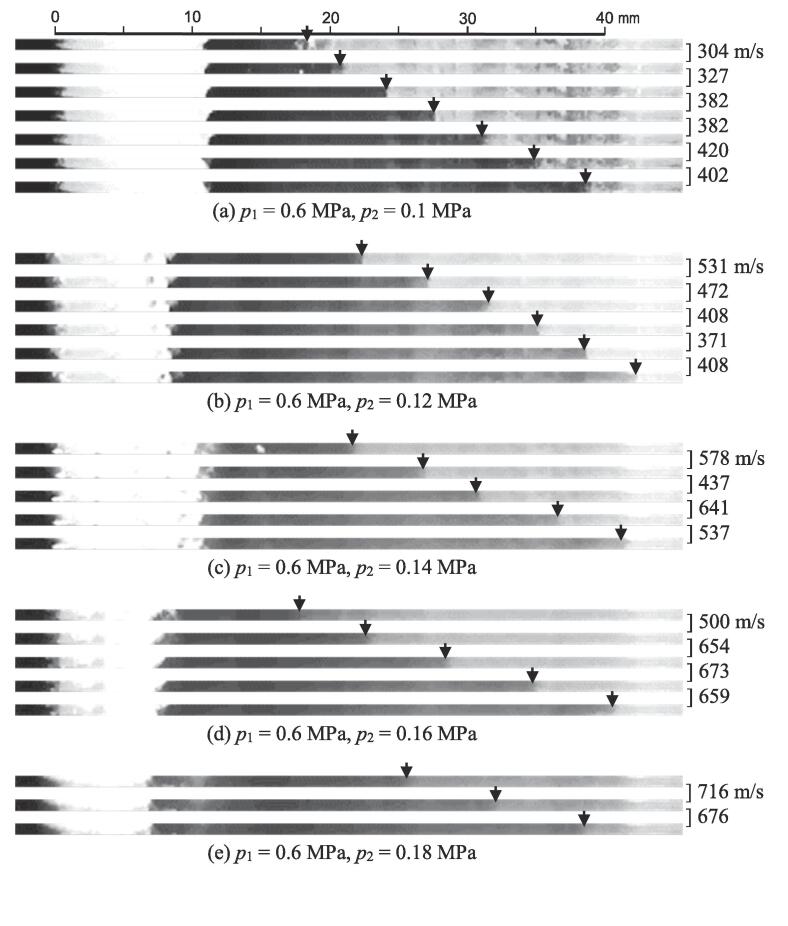
Visualized pressure wave and obtained velocity (a) *p*_1_ = 0.6 MPa, *p*_2_ = 0.1 MPa (b) *p*_1_ = 0.6 MPa, *p*_2_ = 0.12 MPa. (c) *p*_1_ = 0.6 MPa, *p*_2_ = 0.14 MPa. (d) *p*_1_ = 0.6 MPa, *p*_2_ = 0.16 MPa. (e) *p*_1_ = 0.6 MPa, *p*_2_ = 0.18 MPa.

To demonstrate the measurement of the sound velocity, Fig. 9 shows the images taken by the high-speed video camera at 109,999 fps with *p*_2_ at *p*_1_ = 0.6 MPa. As the height of the observation area was limited to increase the recording time, the central region of the Venturi tube was observed. In Fig. 9, the boundary between the black and white regions is indicated by the arrow, and the sound velocity, which was calculated from the distance of the movement of the boundary and the time, is shown on the right-hand side of the images. The sound velocity varied but increased with *p*_2_. When *p*_2_ was increased at constant *p*_1_, *σ* increased. Then, the void ratio decreased with an increase in *σ*. Here, the void ratio is defined as the ratio between the bubble volume and liquid volume. It was reported that the sound velocity changed with the void ratio, and the sound velocity increased with a decrease in the void ratio in a certain void ratio region [55]. When the void ratio is less than 0.1, the sound velocity increases from less than the sound velocity in air to the sound velocity in water as the void ratio decreases [55]. The obtained sound velocity was reasonable.

**Fig. 10:**
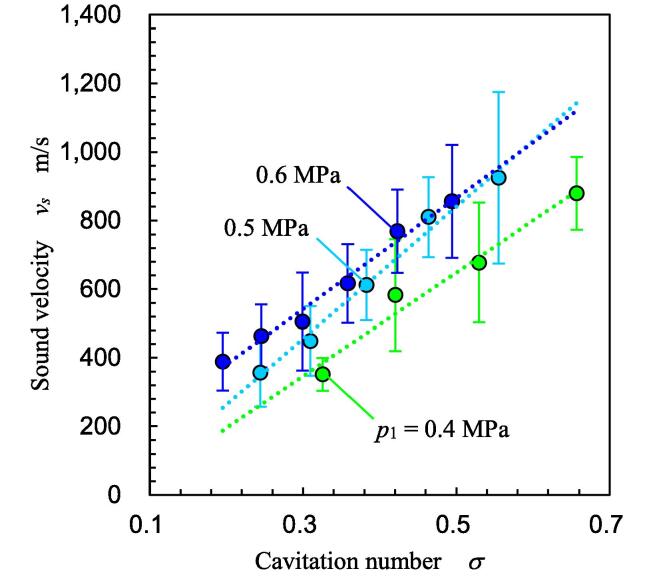
Sound velocity as a function of cavitation number.

Fig. 10 illustrates the sound velocity *v_s_* at various *p*_1_ and *p*_2_ values. Averaged values and standard deviations were obtained from 20 points at each *p*_1_ and *p*_2_. In Fig. 10, *v_s_* is revealed as a function of *σ* because *σ* is the key parameter of the cavitating flow. Because the sound velocity depends on the void ratio in the cavitating flow field and the void ratio changes with time, the standard deviation is large. However, it can be concluded that *v_s_* increases with *σ*. Thus, the experimental formula to estimate *v_s_* from *σ* is as follows:

(3) $$v_{s}=c_{1}\sigma +c_{2}$$

Here, *c*_1_ [m/s] and *c*_2_ [m/s] are constants, and they were 8395 and 469 for *p*_1_ = 0.4 MPa, 7486 and 418 for *p*_1_ = 0.5 MPa, and 4805 and 121 for *p*_1_ = 0.6 MPa, respectively. Thus, *c*_1_ and *c*_2_ can be expressed, respectively, as follows:

(4) $$c_{1}=-88590{p_{1}}^{2}+70639p_{1}-5686$$

(5) $$c_{2}=-12287{p_{1}}^{2}+10549p_{1}-1785$$

In the cavitating flow field in the present experiment, *v_s_* at any *p*_1_ and *p*_2_ can be estimated from Eqs. (1), (3) – (5).

### 3.3 Cavitating length of hydrodynamic cavitation in the Venturi tube

**Fig. 11:**
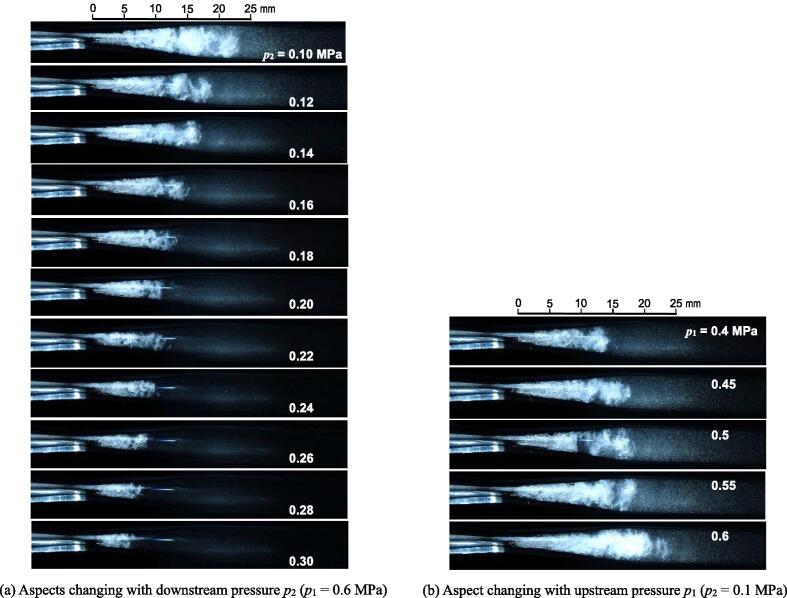
Typical aspects of cavitation in Venturi tube (a) Aspects changing with downstream pressure *p*_2_ (*p*_1_ = 0.6 MPa) (b) Aspect changing with upstream pressure *p*_1_ (*p*_2_ = 0.1 MPa).

Because the aggressive intensity of both hydrodynamic cavitation and ultrasonic cavitation depends on the bubble size, the size of hydrodynamic cavitation in the Venturi tube was considered. Because the diameter of the vortex cavitation is submillimeter, as shown in Fig. 4, and it changes with time drastically, as revealed in Fig. 5, Fig. 6, the size of vortex cavitation was estimated from the cavitating region in the present study. Fig. 11 (a) shows the typical aspect of the hydrodynamic cavitation in the Venturi tube changing with *p*_2_ at *p*_1_ = 0.6 MPa, and Fig. 11 (b) reveals the aspect changing with *p*_1_ at *p*_2_ = 0.1 MPa. As shown in Fig. 5, Fig. 6, the cavitating length changes with time. The longest one was chosen from 20 images, as shown in Fig. 11. As reported elsewhere [7], the cavitating length decreased with an increase in *p*_2_, as shown in Fig. 11 (a). This is because the pressure difference between *p*_1_ and *p*_2_ decreased when *p*_2_ increased. The cavitating length increased with *p*_1_ with constant *p*_2_, as shown in Fig. 11 (b).

**Fig. 12:**
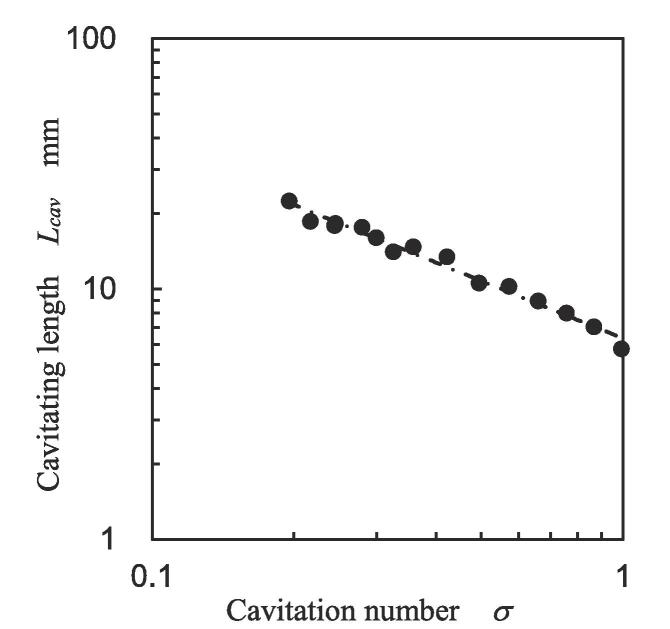
Cavitating length as a function of cavitation number.

The following experimental formula was used to obtain the cavitating length *L_cav_* from *σ* [99], and the cavitating lengths of Fig. 11 are plotted in Fig. 12.

(6) $$L_{\mathrm{cav}}=c_{3}\sigma^{c_{4}}$$

Here, *c*_3_ [mm] and *c*_4_ are constants, and they are 6.358 and − 0.761, respectively. As shown in Fig. 12, all plots are on the line. Then, *L_cav_* at various *p*_1_ and *p*_2_ can be estimated from *σ* by using Eq. (6).

As shown in Fig. 11 (a), when *p*_2_ was increased, the vortex core was clearly observed. The vortex pattern in Fig. 11 (a) suggested that vortex cavitations were combined with each other with shedding downstream, similar to the Karman vortex. This means that the shedding frequency *f_s_* at small *L_cav_* was higher and *f_s_* at large *L_cav_* was lower. This tendency corresponds well to the results of Eq. (2), which means *f_s_* at large *σ* was higher and *f_s_* at small *σ* was lower.

### 3.4 Luminescence intensity of hydrodynamic cavitation in the Venturi tube

**Fig. 13:**
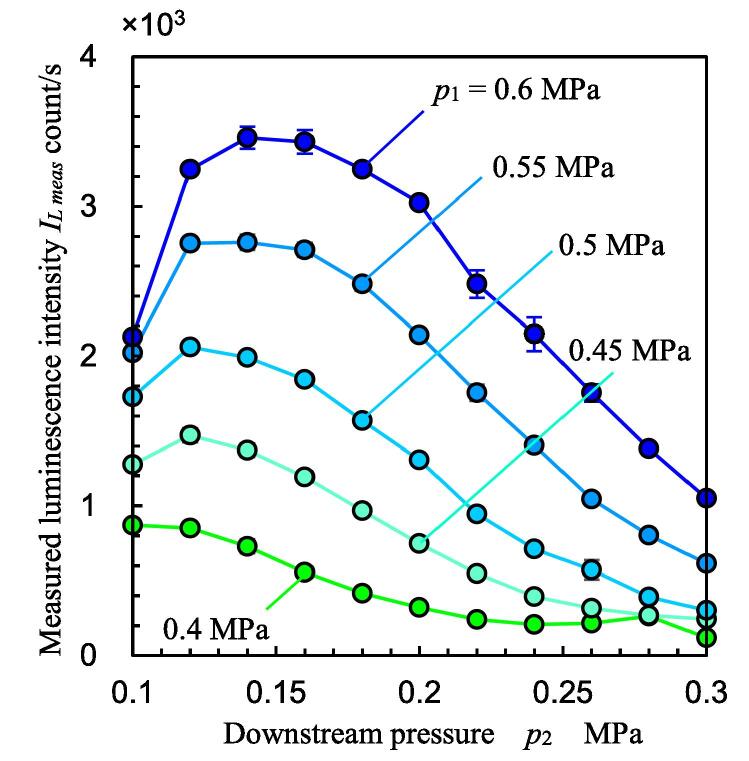
Measured luminescence intensity as a function of downstream pressure.

Fig. 13 illustrates the measured luminescence intensity *I_L meas_* as a function of *p*_2_ at various *p*_1_. As reported previously [7], *I_L meas_* increased with *p*_1_, and it was maximum at a certain *p*_2_ at constant *p*_1_. In the present work, the *p*_2_ in which *I_L meas_* was maximum is denoted by *p*_2 max_. In the present experiment, the throat of the Venturi tube was set 30 mm upstream from the center of the window for the photomultiplier, and the maximum cavitating length was shorter than 30 mm, as shown in Fig. 11, Fig. 12. The setting of the Venturi tube and window for the photomultiplier suggests that the luminescence spots were getting further from the photomultiplier when *p*_2_ was increased. Thus, the setting confirms that the luminescence intensity increases with an increase in *p*_2_ until *p*_2 max_. The *p*_2 max_ changed with *p*_1_ and increased with *p*_1_. For example, *I_L meas_* was (2.127 ± 0.041) × 10^3^ count/s at *p*_1_ = 0.6 MPa and *p*_2_ = 0.1 MPa, and it was (3.459 ± 0.074) × 10^3^ count/s at *p*_1_ = 0.6 MPa and *p*_2_ = 0.14 MPa. In other words, *I_L meas_* had increased by 1.63 ± 0.07 times without an increase in the additional energy for the hydrodynamic cavitation.

**Fig. 14:**
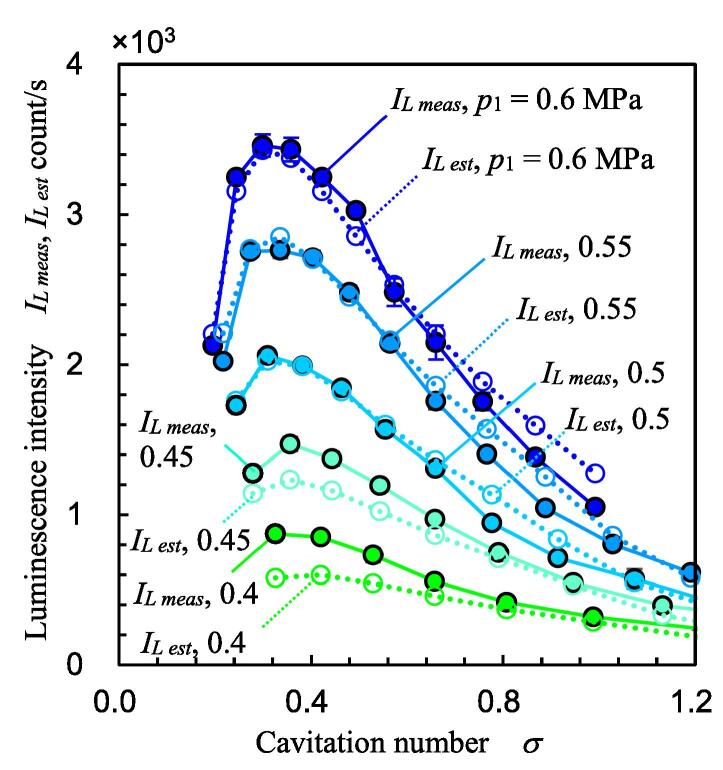
Luminescence intensity as a function of cavitation number.

Fig. 14 illustrates the measured luminescence intensity *I_L meas_* as a function of *σ* at various *p*_1_ by solid lines. The *I_L meas_* was maximum at *σ* ≈ 0.4, as reported previously [7]. Thus, *p*_2 max_ can be estimated by considering *σ*. Because it is organized by the cavitation number, it is a unique phenomenon of the cavitating flow field. The mechanism explaining why luminescence intensity *I_L meas_* was maximum at a certain cavitation number *σ* is discussed in Section 4.

## 4 Discussion

As the luminescent spots induced by the cavitating jet were observed using an EM-CCD camera [81], the luminescence intensity was proportional to the number of luminescent spots per unit time and the individual luminesce intensity. The luminescent spots per unit time can be regarded as the number of vortex cavitations per unit time, and the individual luminesce intensity can be regarded as the individual aggressive intensity, *I_cav_*, of the vortex cavitation collapse. Thus, to estimate the luminescence intensity *I_L est_* under various conditions, it was assumed that *I_L est_* was proportional to the number of the vortex cavitations per unit time and the individual aggressive intensity *I_cav_* of the vortex cavitation collapse. The number of vortex cavitations per unit time is expressed by the shedding frequency of the vortex cavitation, *f_s_*. Thus, *I_L est_* can be described by the product of *I_cav_* and *f_s_*, as follows:

(7) $$I_{Lest}\propto I_{\mathrm{cav}}{∙f}_{s}$$

Because the energy of cavitation is proportional to the volume of the cavitation and the pressure difference of the bubble [100], and it was reported that the number of photons was proportional to the maximum diameter of the bubble [75], *I_cav_* was assumed as follows. The volume of the cavitation was expressed by the cube of the cavitating length, (*L_cav_*)^3^, as follows:

(8) $$I_{\mathrm{cav}}\propto {{(L}_{\mathrm{cav}})}^{3}∙\left(p_{2}-p_{v}\right)$$

On the other hand, as *I_cav_* is affected by the acoustic impedance [50], [51], the term of the sound velocity *v_s_* was added in Eq. (8). Because *I_cav_* was also affected by the flow velocity, which was defined by the pressure difference, i.e., $\sqrt{p_{1}-p_{2}}$, the velocity term was also added in Eq. (8).

(9) $$I_{\mathrm{cav}}\propto {{(L}_{\mathrm{cav}})}^{3}∙\left(p_{2}-p_{v}\right)∙\left(v_{s}-v_{s\;th}\right)∙{\left(\sqrt{p_{1}-p_{2}}\right)}^{n}$$

Here, *v_s th_* is the threshold level of the velocity considering the threshold level of *I_cav_* [101]. The *n* is the exponent in Eq. (9) to consider the power law of the velocity on *I_cav_* [102], [103], [104], [105], [106]. From Eqs. (7), (9), Eq. (10) is obtained.

(10) $$I_{Lest}=c_{5}{{(L}_{\mathrm{cav}})}^{3}∙\left(p_{2}-p_{v}\right)∙\left(v_{s}-v_{s\;th}\right)∙{\left(\sqrt{p_{1}-p_{2}}\right)}^{n}{∙f}_{s}$$

Here, *c*_5_ is constant. As mentioned above, *f_s_*, *v_s_*, and *L_cav_* can be calculated using Eqs. (2), (3), and (6) from *σ*, which is defined by *p*_1_, *p*_2_, and *p_v_*. Moreover, *c*_5_, *v_s th_*, and *n* were obtained by a least-squares method compared with *I_L meas_* and *I_L est_*. In the preset experiment, *c*_5_, *v_s th_*, and *n* were 1.51 × 10^−4^, 190 m/s, and 4.95, respectively. In the present calculation, when *v_s_* obtained from Eq. (3) was larger than 1500 m/s, it was replaced by *v_s_* = 1500 m/s. Then, *I_L est_* can be calculated from the cavitating flow condition, i.e., *p*_1_, *p*_2_, and *p_v_*.

**Fig. 15:**
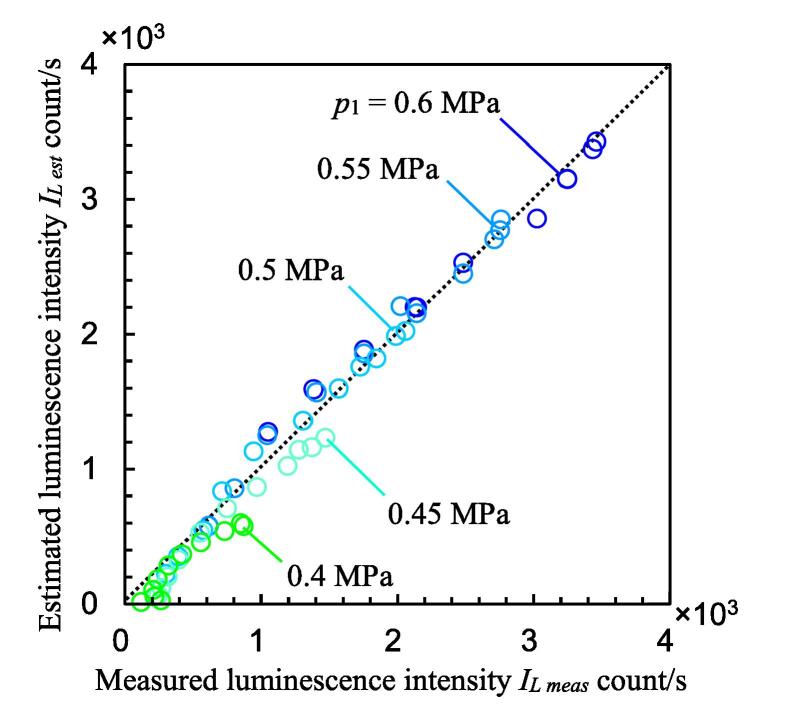
Relationship between measured luminescence intensity and estimated luminescence intensity.

In Fig. 14, *I_L est_* is shown with *I_L meas_*, and *I_L est_* is illustrated by dotted lines. In Eq. (10), (*L_cav_*) and $\sqrt{p_{1}-p_{2}}$ decrease as σ increases. By contrast, (*p*_2_ –*p_v_*), (*v_s_* – *v_s th_*) and *f_s_* increase as σ increases. This is why the luminescence intensity increases up to a certain cavitation number and then decreases. As ${\left(\sqrt{p_{1}-p_{2}}\right)}^{n}$ was in Eq. (10) and *n* = 4.95, the trend stabilized at a lower value of *p*_1_. To investigate the correlation between *I_L meas_* and *I_L est_*, Fig. 15 reveals the relationship between *I_L meas_* and *I_L est_*. As shown in Fig. 14, *I_L est_* describes the peak at *σ* ≈ 0.4 at each *p*_1_. The solid lines, i.e., the measured values, and the dotted lines are very close. As shown in Fig. 14, *I_L est_* of a lower cavitation number at a low upstream pressure is smaller than *I_L meas_*. As shown in Fig. 10, *v_s_* at *p*_1_ = 0.4 MPa is slightly smaller than that of the other values. This would be one of the reasons. The correlation coefficient between *I_L meas_* and *I_L est_* was 0.992. Because the number of datasets was 55, the probability of a noncorrelation is less than 0.01%. If the probability of a noncorrelation is less than 1%, it can be concluded that the relationship is highly significant. Thus, the relationship between *I_L meas_* and *I_L est_* is highly significant, and *I_L est_* can be obtained by Eq. (10).

As described in Eqs. (2), (3), (6), and (10), when *σ* was increased, *L_cav_* and *p*_1_–*p*_2_ decreased. However, *p*_2_–*p_v_*, *v_s_*, and *f_s_* increased with *σ*. If the term *v_s_*–*v_s th_* was not considered, *I_L est_* did not show a maximum value with respect to *σ*. The sound velocity in the cavitating field was a key factor in considering the luminescence intensity of the hydrodynamic cavitation.

## 5 Conclusions

To investigate the mechanism that explains why the luminescence produced by hydrodynamic cavitation in a Venturi tube has a maximum at a certain cavitation number, the luminescence intensity was measured by a luminescence analyzer at various upstream pressures *p*_1_ and downstream pressures *p*_2_ of the Venturi tube by observing the aspect of the hydrodynamic cavitation by high-speed photography. The results obtained are summarized as follows.

(1) The luminescence intensity was increased by optimizing the downstream pressure without increasing the additional energy. Under the present condition, the luminescence intensity increased 1.63 ± 0.07 times at *p*_1_ = 0.6 MPa.
(2) The collapse and rebound of the vortex cavitation in the Venturi tube were observed, and the pressure wave was detected at the collapse of the vortex cavitation.
(3) The sound velocity in the cavitating flow field was evaluated by observing the aspect of the residual bubbles. The sound velocity increased with an increase in cavitation number because of changes in the void ratio.
(4) A method to estimate the luminescence intensity at various *p*_1_ and *p*_2_ was proposed considering the sound velocity, the frequency of the vortex cavitation, and the cavitating length. The estimated luminescence intensity can describe the tendency of the luminescence intensity to change with cavitation number, and it had a good correlation with the measured luminescence intensity.
(5) The key factors affecting the change in the luminescence of the hydrodynamic cavitation with cavitation number are the vortex cavitation and the sound velocity in the cavitating flow field.

## CRediT authorship contribution statement

**Hitoshi Soyama:** Conceptualization, Data curation, Formal analysis, Funding acquisition, Investigation, Methodology, Project administration, Supervision, Validation, Visualization, Writing - original draft, Writing - review & editing.

## Declaration of Competing Interest

The authors declare that they have no known competing financial interests or personal relationships that could have appeared to influence the work reported in this paper.
